# Dynamic Recruitment of Protein Tyrosine Phosphatase PTPD1 to EGF Stimulation Sites Potentiates EGFR Activation

**DOI:** 10.1371/journal.pone.0103203

**Published:** 2014-07-25

**Authors:** Pedro Roda-Navarro, Philippe I. Bastiaens

**Affiliations:** 1 Department of Immunology, School of Medicine, Complutense University and ‘12 de Octubre’ Health Research Institute, Madrid, Spain; 2 Department of Systemic Cell Biology, Max Planck Institute of Molecular Physiology, Dortmund, Germany; Hungarian Academy of Sciences, Hungary

## Abstract

Balanced activity of protein tyrosine kinases and phosphatases (PTPs) controls tyrosine phosphorylation levels and, consequently, is needed to prevent pathologies like cancer. Phosphatase activity is tightly regulated in space and time. Thus, in order to understand how phospho-tyrosine signalling is regulated, the intracellular dynamics of PTPs should be investigated. Here, we have studied the intracellular dynamics of PTPD1, a FERM (four-point-one, ezrin, radixin, moesin) domain-containing PTP that is over expressed in cancer cells and potentiates EGFR signalling. Whereas PTPD1 was excluded from E-cadherin rich cell-cell adhesions in epithelial cell monolayers, it diffused from the cytoplasm to those membranes in contact with the extracellular medium. Localisation of PTPD1 at the plasma membrane was mediated by its FERM domain and enabled the formation of EGFR/PTPD1-containing signalling complexes that pre-existed at the plasma membrane before EGF stimulation. PTPD1 and EGFR transiently co-localised at EGF stimulation sites until the formation of macropinosomes containing active species of EGFR. Interference of PTPD1 expression caused a decrease in EGFR phosphorylated species at the periphery of the cell. Presented data suggest that the transient formation of dynamic PTPD1/EGFR signalling complexes strengthens EGF signalling by promoting the spatial propagation of EGFR phosphorylated species.

## Introduction

Receptor tyrosine kinases (RTKs) regulate cell differentiation, proliferation, survival and motility. Although these cellular processes govern the normal development of organisms, they are also critical to cancer initiation and progression. Dephosphorylation of RTKs by protein tyrosine phosphatases (PTPs) contributes to terminate extracellular signals triggered by growth factors and hormones and keeps physiological levels of active receptors [1]. This regulatory role of PTPs makes them to be considered as potential tumour suppressors. However, PTPs also function as positive regulators of RTK signalling and oncogenic functions have been also proposed. Although many of the oncogenic PTPs are over expressed in different tumours, their regulatory role on RTK signalling and cancer progression is not completely known [2]. Activity of PTPs is restricted in space and time [3]–[5]. Thus, in order to fully understand the spatio-temporal regulation of RTK by PTPs, it is needed to probe their intracellular dynamics in live cells, the natural environment that confers spatial and temporal confinements to protein functions.

PTPD1 [6], a classical Four point one, Ezrin, Radixin, Moesin, (FERM) domain-containing PTP, has been shown to potentiate EGF signalling in primary fibroblast [7] and bladder tumour cells [8]. Although PTPD1 is over expressed in tumour cells, its intracellular dynamics during EGF stimulation has not been studied. Here we have tracked the intracellular dynamics of PTPD1 in cells derived from breast cancer, an example where EGF signalling is linked to carcinogenesis [9]. We have found that accessibility of PTPD1 to the plasma membrane is mediated by the FERM domain and restricted by E-cadherin rich cell-cell adhesions found in epithelial monolayers. Analysis of protein dynamics demonstrates the diffusion of PTPD1 from the cytoplasm to the plasma membrane. This process enables the co-localisation of PTPD1 and EGFR in EGF-induced endocytic structures, in which the phosphatase stays until the complete formation of active EGFR-containing macropinosomes. Dynamic PTPD1/EGFR-containing signalling complexes are formed even before EGF stimulation as demonstrated by fluorescence cross-correlation spectroscopy (FCCS). Interference of PTPD1 expression suggests that these signalling complexes are needed for the spatial propagation of EGF-induced EGFR phosphorylated species. Thus, we propose that the regulated accessibility of PTPD1 to dynamic EGFR-containing signalling complexes at the plasma membrane potentiates early EGF signalling by promoting the propagation of active species of EGFR.

## Results

### Diffusion of PTPD1 to the plasma membrane is prevented by cell-cell adhesions

**Figure 1:**
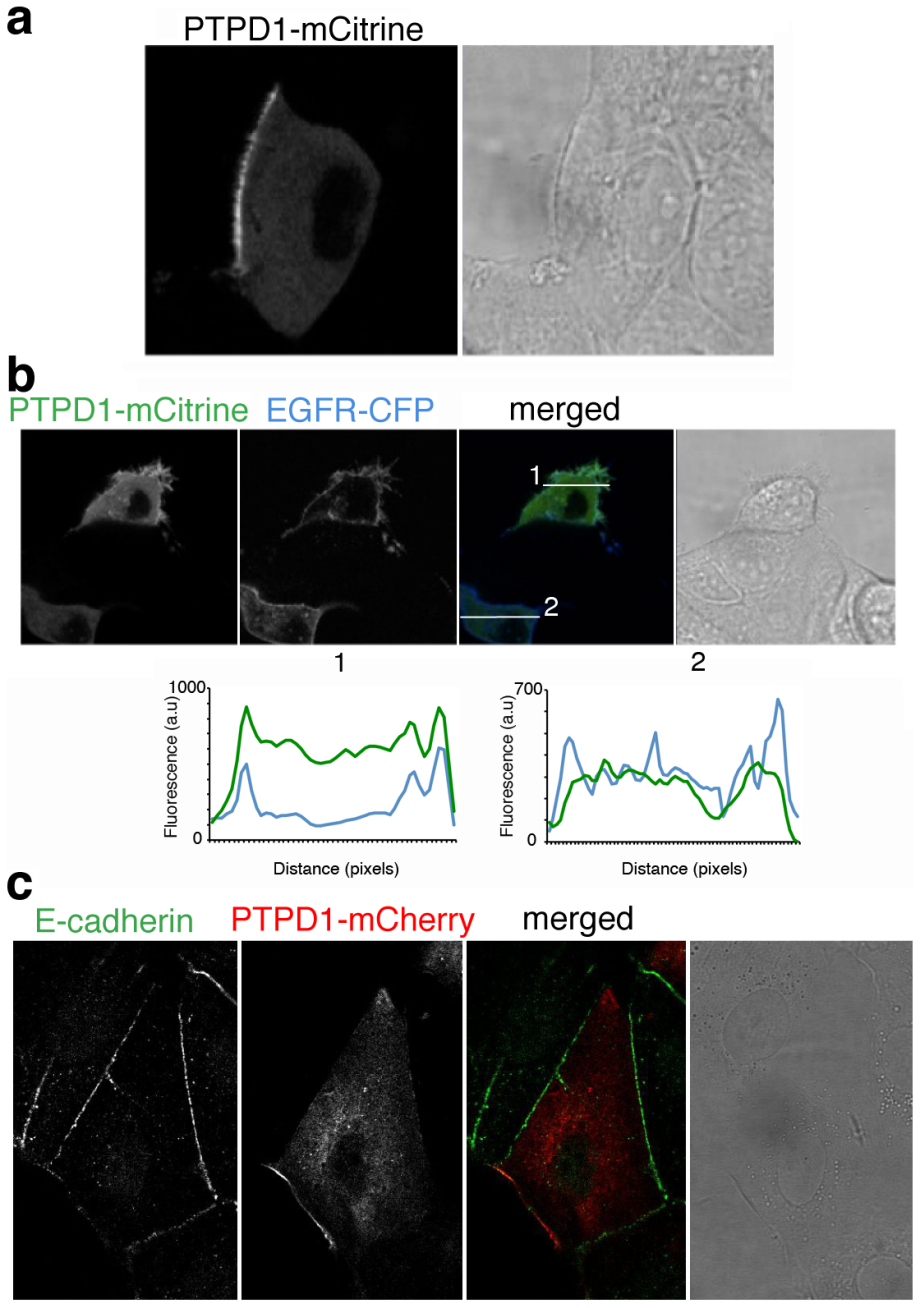
Exclusion of PTPD1 from the E-cadherin mediated cell-cell adhesions in epithelial cell monolayers. (**a**) Steady state expression of PTPD1-mCitrine fusion protein in MCF7 cells. The mCitrine channel and the light transmission of a confocal section are shown. (**b**) Distribution of PTPD1-mCitrine and EGFR-CFP fluorescent fusion proteins in MCF7 cells. mCitrine and CFP channels, the merged image, and the light transmission of a confocal section are shown. White lines mark the places of cells where fluorescence intensity profiles 1 and 2 where done (lower graphs). The co-localisation of both proteins is only observed in membrane structures of the cell placed at the edged of the monolayer (**c**) Distribution of PTPD1-mCherry fluorescent fusion protein and endogenous E-Cadherin. Green (E-Cadherin), red (PTPD1), merged channels and transmission light of a confocal section are shown. The staining shows the exclusion of PTPD1 from E-Cadherin rich sites.

**Figure 2:**
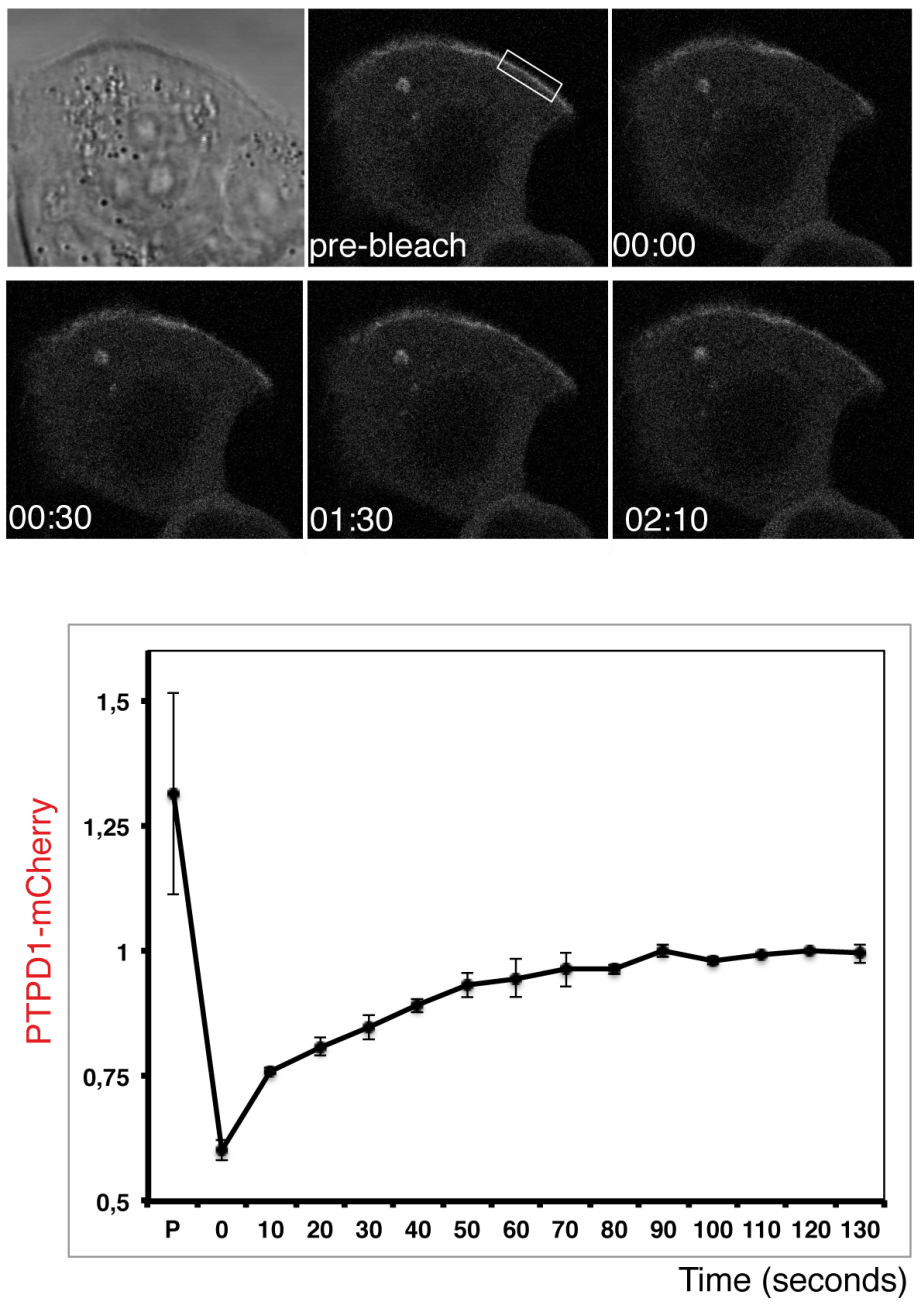
Dynamic replenishment of PTPD1 at the plasma membrane assessed by FRAP. The fluorescence of PTPD1-mCherry before bleaching and in frames obtained at several time points after bleaching are shown (Upper panels). Numbers indicate the corresponding time of the frame (minutes:seconds). The transmission light image is shown. The bleached area is indicated with a square in the image that shows the cell before bleaching. Lower graph shows the mean and the standard deviation of recovery curves of the normalised fluorescence in 3 representative cells. FRAP data is obtained as explained in material and methods, and these data are normalised to the maximum fluorescence value recovered after bleaching. Fluorescence recovery indicates that diffusing cytoplasmic protein reaches the plasma membrane.

The regulatory role of PTPD1 on EGF signalling [7], and the pathological role of over expressed EGFR during breast carcinogenesis and metastasis [9], makes breast cancer an interesting model for studying PTPD1 dynamics. Experiments were implemented with the breast cancer-derived cell line MCF7, which share genomic and transcriptional features with primary tumour cells [10]. Steady state distribution of a PTPD1-mCitrine chimera was studied in live cells under growing conditions. PTPD1 was distributed in the cytoplasm and excluded from the plasma membrane at cell-cell adhesion sites of cells embedded in cell monolayers. The phosphatase was nonetheless accumulated at free plasma membranes (**Fig. 1a**) where it co-localises with an EGFR-CFP chimera (**Fig. 1b**). Staining for E-cadherin demonstrated the exclusion of PTPD1 from the E-cadherin rich cell-cell contacts (**Fig. 1c**). These data suggested that PTPD1 is dynamically recruited to free plasma membranes from diffusing cytoplasmic pool of protein. To test this hypothesis fluorescence recovery after photobleaching (FRAP) experiments were done (**Fig. 2**). PTPD1 at the plasma membrane was bleached and the replenishment of fluorescent protein along time was measured. Fluorescence rapidly recovered with a half-time (*t*_1/2_) of around 10–15 seconds, suggesting that diffusing cytoplasmic protein reaches the plasma membrane. An immobile fraction of PTPD1 was deduced from the existence of irreversible photobleached protein, suggesting the existence of PTPD1 interacting molecules at the plasma membrane. Together these results strongly suggest a regulatory mechanism for the exclusion of diffusing PTPD1 from the E-cadherin mediated cell-cell adhesions in MCF7 monolayers. The phosphatase seems to be nonetheless available at free plasma membranes to participate in EGF signalling.

### Transient location of PTPD1 to EGF stimulation sites during early EGF signalling

Enhanced EGF signalling is associated with a reduction in E-cadherin mediated adhesion [11], [12]. Thus, to further study the dynamics of PTPD1 and EGFR at the plasma membrane we used MCF7 cells stably transfected with an expression vector coding for EGFR coupled to the green fluorescent protein (GFP) (called MCF7-EG). Initially the specific binding of EGF to the ectopically expressed EGFR-GFP in these cells was proved. The expression levels of EGFR-GFP in MCF7-EG cells were comparable to endogenous EGFR levels in the breast-cancer derived cell line MDA-MB-468 (**Fig. S1a**). Moreover, EGF stimulation induced a sustained EGFR tyrosine phosphorylation in both cell lines (**Fig. S1b**). Therefore, over expression of EGFR in MCF7 cells reproduced the EGF-induced sustained phosphorylation of EGFR found in epithelial cancer cells that express unusual high levels of the receptor [13], [14].

**Figure 3:**
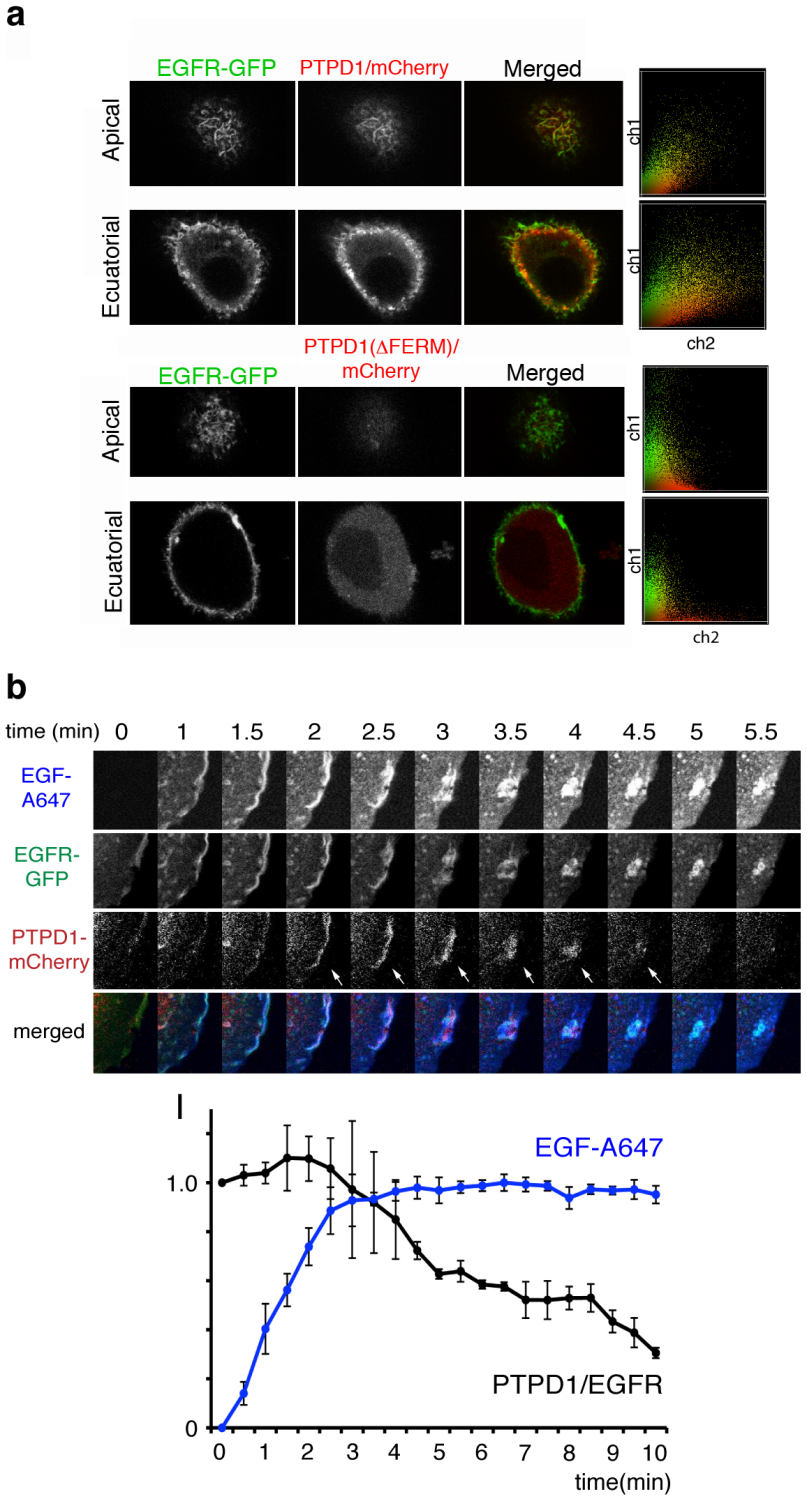
Distribution of PTPD1 in EGFR overexpressing cells. (**a**) Distribution of PTPD1-mCherry (upper panels) and the mutant PTPD1(ΔFERM)-mCherry (lower panels) at membrane ruffles in MCF7-EG cells. Apical and equatorial confocal sections are shown. Right panels show co-localisation histograms. (**b**) Detail of frames of the ROI marked in the time-lapse microscopy experiment shown in Figure S2 and Movies S1 and S2. The dynamic distribution of EGFR-GFP, PTPD1-mCherry and EGF-A647 during EGF-induced endocytosis of membrane ruffles is shown. Lower graph shows the intensity (*I*) of the EGF-A647 (blue) normalised to the maximum value, and the ratio PTPD1-mCherry/EGFR-GFP normalised to the value at the time point zero. Average values and standard deviation of 4 endocytic structures obtained from 2 cells are represented. Data suggest the FERM domain-mediated transient distribution of PTPD1 to early EGF stimulation sites.

PTPD1 contains a FERM (four point one, ezrin, radixin, moesin) domain for the binding of phosphoinositides at the plasma membrane [15]. Then we initially wanted to test whether the FERM domain could mediate the distribution of PTPD1 to EGFR sites at the plasma membrane. To do these experiments we used PTPD1 and a PTPD1(ΔFERM) mutant coupled to the fluorescent protein mCherry. Steady state distribution of these PTPD1 chimeras revealed a clear-cut co-localisation of wild type PTPD1 and EGFR in apical and equatorial membrane ruffles of cells. Consistent with the capacity of FERM domains to bind the plasma membrane, localisation of PTPD1 at membranes was dependent on the FERM domain as demonstrated by the homogeneous cytoplasmic distribution of the PTPD1(ΔFERM)-mCherry mutant (**Fig. 3a**). These data suggested that the binding of PTPD1 to the plasma membrane enabled the formation of PTPD1/EGFR signalling complexes.

Then we wanted to assess the dynamic distribution of PTPD1 during early EGF stimulation. Fluorescently labelled EGF, EGFR-GFP, and PTPD1-mCherry were simultaneously tracked in time-lapse confocal microscopy. PTPD1 co-localised with EGFR in lamellipodia at the periphery of starving cells before EGF stimulation (**Fig. 3b**, time 0 minutes, and **Fig. S2**). A transient location of PTPD1 in EGF-induced membrane ruffles during receptor endocytosis was observed. PTPD1 stayed in endocytic structures until the complete formation of macropinosomes containing active species of EGFR, where the phosphatase was no longer detected (**Fig. 3b**, see arrows and **movies S1** and **S2**). PTPD1 also co-localised with cortical actin (**Fig. S3a**) that also assembled and disassembled concomitantly to the EGF-induced endocytic process (**Fig. S3b** and **movie S3**). These data suggest a cycle in which PTPD1 reaches the membrane, participates in actin and membrane dynamics during early EGF signalling, and then diffuses back to the cytosol.

### PTPD1 and EGFR dynamic complexes pre-exist before growth factor stimulation

**Figure 4:**
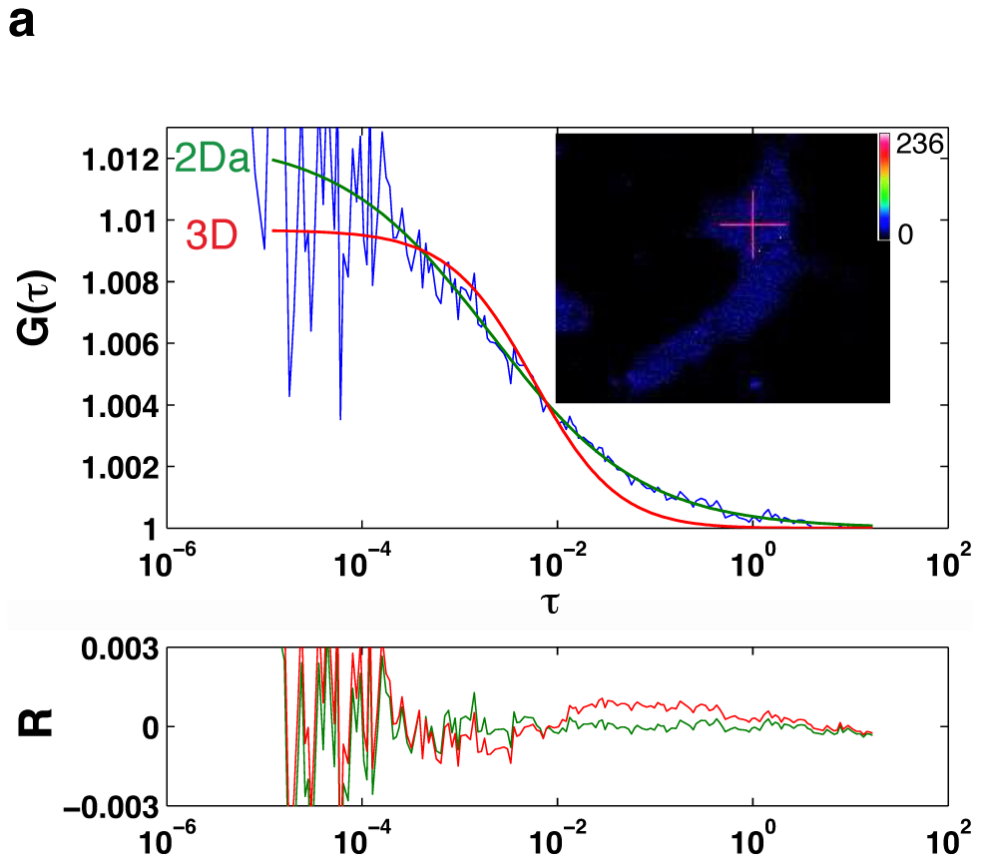
Theoretical models to fit experimental autocorrelation data of PTPD1-GFP obtained by FCS. The upper panel shows the experimental autocorrelation function (*G*(*τ*)) (blue line), and fits of data to the theoretical three-dimensional (3D) or anomalous two-dimensional (2Da) models (red and green lines, respectively). *τ* indicates the delay time in seconds. The lower panel shows residuals (*R*) of each fit (red and green lines). Fits of data indicate an anomalous two-dimensional Diffusion of PTPD1. This model is used to obtain the translational diffusion (τ_d_) and to calculate the diffusion coefficient as explained in *Methods S1*. The inset shows the cell analysed, in which a red cross points the position of the confocal volume. Calibration bar of the fluorescence intensity is shown.

We aimed to further prove the existence of dynamic signalling complexes containing PTPD1 and EGFR by fluorescence cross-correlation spectroscopy (FCCS). Experimental autocorrelation functions (ACFs) of PTPD1-GFP were initially acquired. A 3D free diffusion model could not describe the obtained ACFs. Given the co-localisation of PTPD1 with sub membranous filamentous actin (**Fig. S3a**), and the flat morphology of the studied cells, we test an anomalous 2D diffusion model [16]. This model fitted well the experimental ACFs, consistent with diffusion of PTPD1 at a crowded microenvironment [16] (**Fig. 4**). PTPD1-GFP diffusion was parameterized by a translational diffusion and an anomalous exponent of *τ_d_* = 2.1±1.1 ms and *a* = 0.69±0.13, respectively (n = 9). Fusion of PTPD1 to mCherry did not significantly affect these parameters (*τ_d_* = 2.4±0.78 ms and *a* = 0.63±0.07) (n = 7). Diffusion coefficients for each fluorescent fusion protein were calculated (*D*_PTPD1-GFP_ = 2.5±1.16 µm^2^/s*^a^* and *D*_PTPD1-mCherry_ = 1.9±0.46 µm^2^/s*^a^*).

**Figure 5:**
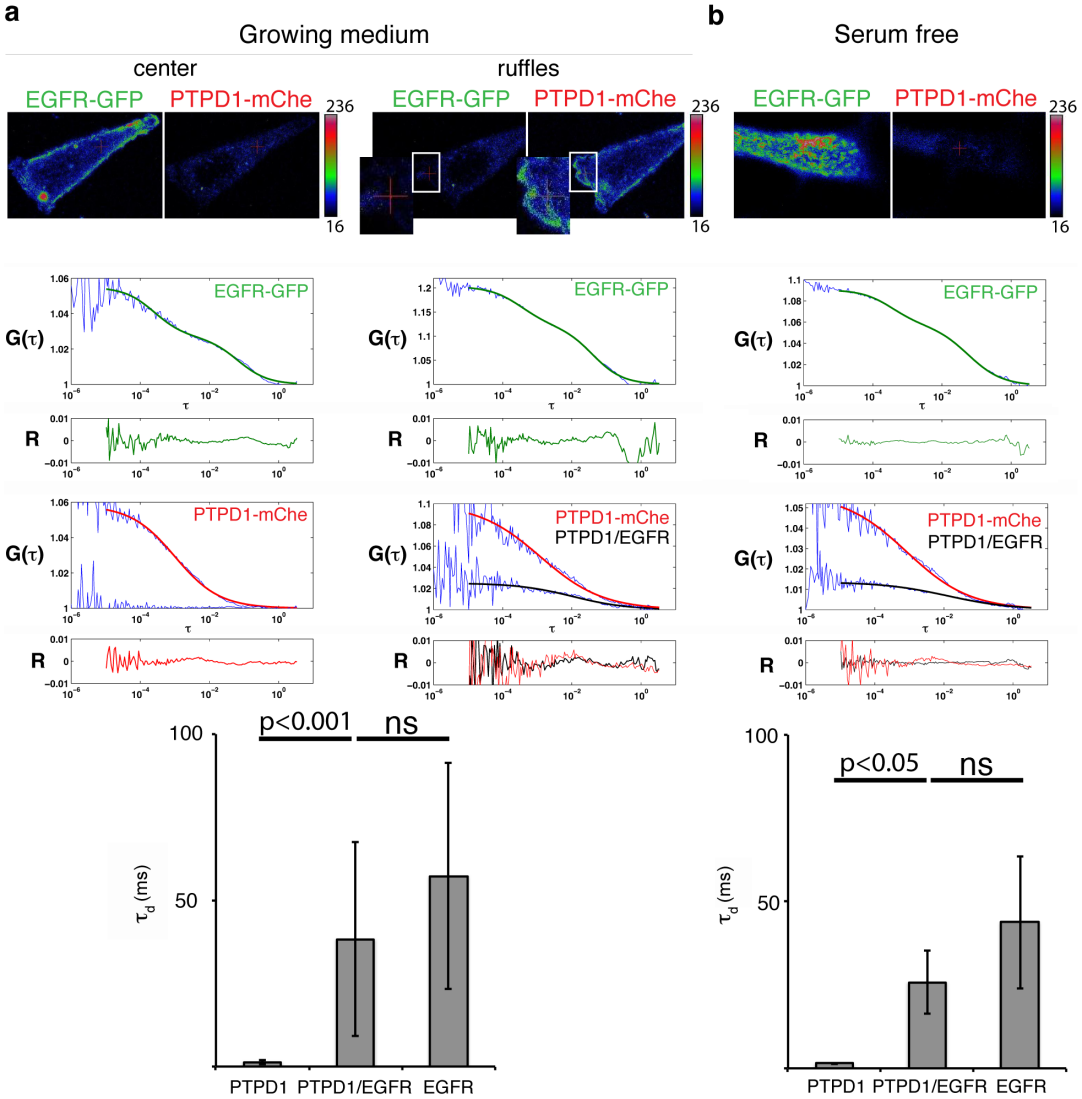
PTPD1 and EGFR containing signalling complexes precede EGF stimulation. (**a** and **b**) FCCS data of PTPD1-mCherry and EGFR-GFP in MCF7-EG cells. Images of MCF7-EG cells transfected with PTPD1-mCherry are shown. Red and green channels are shown for each analysed condition. A red cross points the position of the confocal volume. A magnified area with the position of the confocal volume is shown in ruffles of cells observed under growing conditions. Calibration bars of fluorescence intensity are shown. Graphs under the images represent the experimental autocorrelation and cross-correlation functions (blue lines) as well as the theoretical fits for EGFR, PTPD1 and cross-correlation data in green, red and black lines, respectively. Residuals (*R*) of each fit are shown under each correlation function. *τ* indicates the delay time in seconds. Lower graphs show the average value and standard deviation of the translational diffusion in milliseconds (*τ_d_* (ms)) obtained for PTPD1, the cross-correlation function, which represents PTPD1/EGFR complexes, and EGFR, in growing conditions (**a**, lower graph, n = 6 cells) and serum free conditions (**b**, lower graph, n = 3 cells). The p value of a Mann-Whitney test is shown (ns: non significative; p>0.05) Cross-correlation strongly suggests the formation of dynamic complexes containing PTPD1 and EGFR.

Diffusion properties of PTPD1 and EGFR were then investigated by FCCS in MCF7-EG cells transiently transfected with the PTPD1-mCherry. A substantial overlap of the green and red confocal volumes was proved by using a fluorescent fusion protein containing p38 along with GFP and mCherry at N- and C- terminus (**Fig. S4**). In each cell, GFP and mCherry fluorescence emissions were measured in confocal volumes placed either close to bottom, at the centre of the cell, or at EGFR-containing membrane ruffles (**Fig. 5a**, confocal volume indicated by a red cross). This generated an internal control (the cell centre) in each analysed cell where co-diffusion of both proteins is expected to be less in comparison with data obtained at EGF stimulation sites. Cross-correlation curves were clearly detected in membrane ruffles of cells measured in growing medium, i.e. under growth factor stimulation (**Fig. 5a**, black line fit in right graphs). However, they were barely detected at the centre of cells (**Fig. 5a**, left graphs). Cross-correlation curves were also detected in cells culture in serum free medium (**Fig. 5b**). These data are consistent with the co-localisation of PTPD1 and EGFR observed in pre-stimulated cells (**Fig. 3b** and **Fig. S2**). ACFs of EGFR-GFP showed the existence of two diffusion times consistent with previous observations [17] (**Fig 5a and 5b**, upper graphs). These curves were well described by a theoretical model in which the fast component followed a 3D free diffusion (*τ_d1_* = 0.4±0.1 ms) and the slow component a 2D anomalous diffusion typical of transmembrane proteins (*τ_d2_* = 53±36 ms and *a* = 0.9±0.12) (n = 29). Consistent with the formation of dynamic PTPD1/EGFR-containing signalling complexes, the translational diffusion of cross-correlation curves increased around one order of magnitude with respect to the translational diffusion of free PTPD1-mCherry, matching the magnitude of the translational diffusion of the slow component present at the ACFs obtained for EGFR-GFP (**Fig. 5a and b**, lower graphs). Together, these data strongly supported the notion that dynamic EGFR/PTPD1-containing signalling complexes pre-existed before growth factor stimulation.

### Role of PTPD1 in the spatial regulation of early EGFR phosphorylation

**Figure 6:**
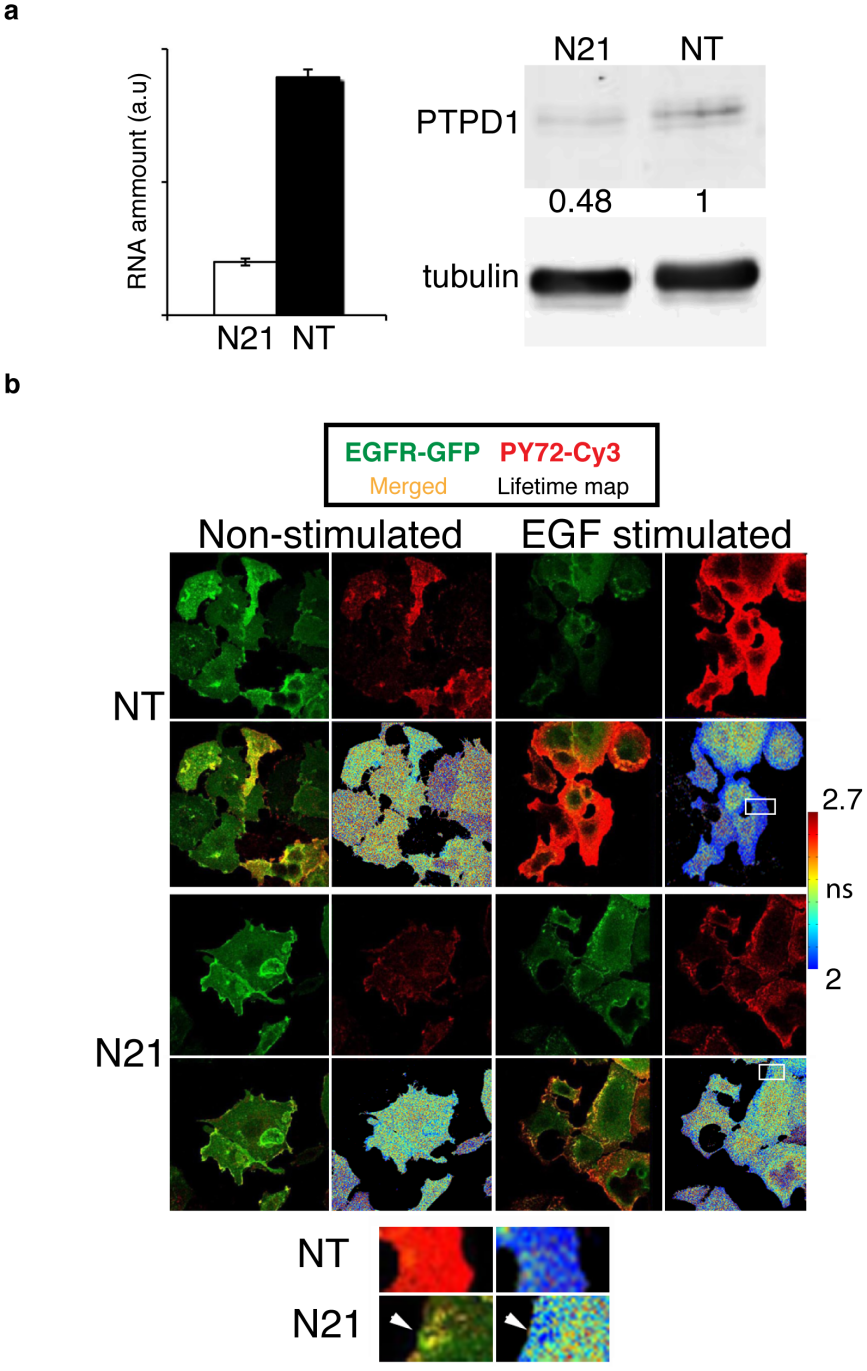
Role of PTPD1 in the spatial regulation of EGFR phosphorylated species (a) Quantitative PCR (left bar graph) and western blot (right panels) to evaluate the modulation of PTPD1 caused by the siRNA N21 transfection. Quantification of the extent of protein down modulation is shown. (b) FLIM to assess the phosphorylation of EGFR in MCF7-EG cells non-stimulated or stimulated 5 min with EGF and transfected with N21 or non-targeting (NT) siRNA pools. The GFP and phospho-tyrosine fluorescence, as well as maps of the GFP lifetime of a confocal section are shown in each condition. Lower panels show zooms of the areas labelled with white squares placed in lifetime maps. Arrowheads point discrete areas of EGFR phosphorylation. Calibration bar of the GFP fluorescence lifetime in nanoseconds (ns) is shown. EGFR-GFP phosphorylation is indicated by the decrease in the fluorescence lifetime of the GFP.

It has been shown that PTPD1 potentiates EGF signalling [7], [8]. However, the regulatory role of this PTP on the dynamics of EGFR activation has not been studied. To investigate this issue the expression of PTPD1 in MCF7-EG cells was interfered by siRNA, and EGF-induced phosphorylated species of EGFR-GFP were imaged in confocal sections by Förster resonance energy transfer-fluorescence lifetime imaging microscopy (FRET-FLIM) as previously described [18]. Transfection of the specific siRNA for PTPD1 (N21) provoked a decrease around 75% and 50% of mRNA and protein levels, respectively (**Fig 6a**). The fluorescence lifetime of the GFP coupled to EGFR was imaged after N21 or non-targeting siRNA (NT) treatment (**Fig. 6b**). 5 minutes EGF stimulation of MCF7-EG induced a strong phosphorylation of the receptor in cells transfected with NT siRNA as revealed by the decreased average lifetime observed in the lifetime map. Low average lifetimes evenly found at the periphery of the cell are consistent with a fast propagation of EGFR phosphorylated species, which we have previously shown after local EGF stimulation [18]. By contrast, in samples transfected with N21 siRNA only discrete areas of phosphorylated receptor were found at the periphery of cells (**Fig. 6b**, magnified areas). Thus, these data suggest that PTPD1 is necessary for the early spatial propagation of EGF-induced EGFR phosphorylated species.

**Figure 7:**
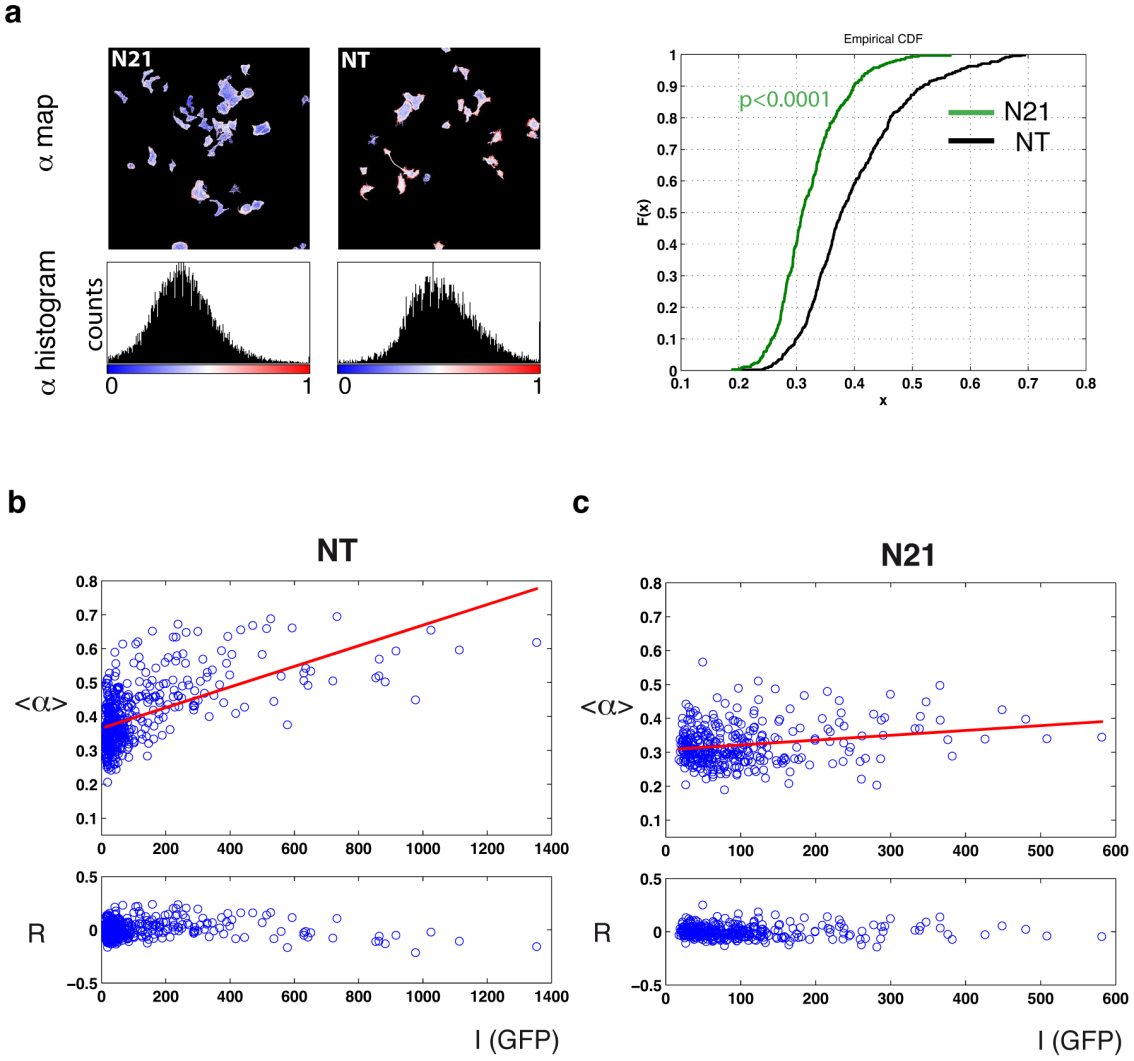
PTPD1 potentiates EGFR phosphorylation. (**a**) Quantification of phosphorylated fraction (*α*) calculated by global analysis of FLIM data. *α*-map (upper) and *α*-histogram (lower) of a representative field of cells transfected with N21 or NT siRNA pools. Calibration bars of *α* values are shown under histograms. The right graph contains the cumulative distribution of average values of *α* obtained for each cell (x = <*α*>) (n = 151 for NT samples and n = 94 for N21). The Kolmogorov-Smirnov test for two samples was used to compare datasets (p value is indicated). (**b** and **c**) Correlation of the *α* value and the expression levels of EGFR-GFP in single cells (blue spots) of samples transfected with the NT (b) or the N21 (c) siRNA pools. Upper graphs show linear fits of data and lower graphs show the residuals (*R*) of fits. Expression levels of EGFR-GFP are estimated by the fluorescence intensity (*I*) of the GFP. Calculation of *α* from FLIM data shows a reduction of the <*α*> per cell in N21 samples. Positive correlation between *I* and *α* is indicative of signal propagation, which would be abolished by PTPD1 down-modulation.

We calculated the molar fraction of phosphorylated EGFR (*α*) per cell by global analysis of time correlated single photon counting (TCSPC) FLIM data in the complex plane [19]. A reproducible decrease in the average value of *α* per cell was observed in those samples in which PTPD1 expression was interfered by N21 siRNA (**Fig 7a**). Thus PTPD1 supports the activation of EGFR in these cells. During early EGF stimulation, PTP activity, such us that of PTP1B, is low at the periphery of the cell [4], enabling the full activation of EGFR. In this scenario, spatial propagation of the signal, promoted by the autophosphorylation of EGFR, would be favoured by high density of receptors. This positive feedback loop in EGFR kinase activation can be promoted by generic inhibition of PTP action [20]. Consistent with this, we found a clear positive correlation between the value of *α* and the expression levels of EGFR, assessed by the GFP intensity (**Fig. 7b**). Down-modulation of PTPD1 by N21 siRNA abolished this correlation (**Fig. 7c**). These data, supports the notion that PTPD1 assists in the spatial propagation of EGFR phosphorylated species and, consequently, enhances EGFR activation in these cells.

## Discussion

A positive role of certain PTPs on RTK signalling and cancer progression has been established. It is nonetheless not completely understood how RTKs are regulated by oncogenic PTPs over expressed in transformed cells [8], [21]–[23]. Given the spatial and temporal tight regulation of the function of PTPs [24], their intracellular dynamics should be investigated. We have tracked the dynamics of PTPD1, a positive regulator of EGF signalling over expressed in cancer cells [8], [25]. Our data show that regulated diffusion of PTPD1 to the plasma membrane enables the formation of dynamic signalling complexes with EGFR. Moreover, evidences are contributed suggesting that this dynamics is necessary for the spatial propagation of EGFR phosphorylated species during early EGF signalling.

Live cell-imaging (steady state and FRAP experiments) shows that PTPD1 diffusion to the plasma membrane is restricted by E-cadherin cell-cell adhesions (**Fig. 1** and **2**). Diffusion speed of PTPD1, as deduced from the *t*_1/2_ found in FRAP experiments, is similar to other cytoplasmic PTPs [26]. Immobile fractions found in this work can be caused by a slow off-rate from binding sites at the plasma membrane or simply by limited recovery due to variable cell size. FCS accurately calculated the diffusion coefficient of PTPD1 (*D*_PTPD1_). Taken into account the anomalous exponent (*a*), our data indicate a *D*_PTPD1_ of around 1 µm^2^/s. The 2D anomalous diffusion found in our analysis (**Fig. 4**) is consistent for proteins interacting with other cellular components in the crowded environment of the cytoplasm of flat cells. In this regard, the FERM domain of the protein has been show to mediate the plasma membrane location of the PTP (**Fig. 2**), where it is in close proximity with the actin cytoskeleton (**Fig. S3a**), as has been also found by other authors [27].

In individual cells, out of monolayers, PTPD1 accumulates at the plasma membrane and transiently stays at EGF-induced membrane ruffles, disappearing from the endocytic structure just before the formation of active EGFR-containing macropinosomes (**Fig. 3b**). Transient enrichment of phosphatidylinositol (4, 5) bisphosphate (PIP_2_) at membrane ruffles during growth factor stimulation [28] and the ability of FERM domains to bind PIP_2_ [29] supports the idea that PIP_2_ enrichment could favour the FERM-dependent PTPD1 distribution to membrane ruffles found in our work. It is tempting to speculate that the peak of PIP_3_ concomitant to macropinosome formation [28] would mediate the observed release of PTPD1 to the cytoplasm (**Fig. 3b**). Since dynamic local homeostasis of PIP_2_ is crucial for cyclical regulation of cytoskeleton and vesicle movement [30], PTPD1 could have a regulatory role in acting polymerization cycles needed for the correct membrane dynamics occurring just after EGF stimulation. Consistent with this idea PTPD1 seems to be required for the stability of actin filaments [27].

Although, steady state distribution of PTPD1 to actin filaments and adhesion plaques has been previously observed in primary fibroblasts [27], our data show that in cancer cells that over expressed PTPD1, EGFR/PTPD1-containing signalling complexes could be formed at membrane ruffles before EGF stimulation (**Fig. 5b**). Although co-diffusion of PTPD1 and EGFR observed in FCCS does not mean a direct physical interaction, it demonstrates the coincidence in time of proteins in supra molecular structures. FRET or pull down experiments did not show a direct interaction between EGFR and PTPD1 (not shown). Thus, PTPD1/EGFR-containing signalling complexes observed at the cell periphery should be formed by the interaction of PTPD1 with other proteins or membrane lipids rather than direct physical interactions with EGFR.

Interfering PTPD1 expression suggests that the protein is involved in propagating phosphorylated species of EGFR, and that this is needed to reach the optimal activation of the receptor in the cell (**Fig. 6** and **7**). It is not clear at this step what is the exact role of PTPD1 at these sites. One possibility is that it can raise c-SRC activity, as previously described [7]. PTPD1, EGFR and c-SRC could then regulate actin and membrane dynamics needed to propagate EGF signals and consequently promote cancer progression. In this regard, activation of EGFR and c-SRC appear to contribute to aggressive phenotypes of human tumors [31]. Although studies by Cardone and co-workers [7] suggest a role of PTPD1 phosphatase activity in c-SRC activation, recent studies demonstrated that PTPD1 has no catalytic activity *in vitro* [32]. Thus, we cannot discard the possibility that the function of PTPD1 in propagating EGFR phosphorylated species is not dependent on its phosphatase activity. Functions of PTPs not dependent on the catalytic activity have been shown [33].

Our data suggest a model in which diffusing PTPD1 is bound by its FERM domain to the plasma membrane facing the extracellular medium, and then incorporated along with EGFR in signalling complexes. The transient location of PTPD1 in these signalling complexes during EGF stimulation might be important for cytoskeleton and membrane dynamics concomitant to EGFR endocytosis. Once macropinosomes are formed and the PTP is no longer needed, it is released to the cytoplasm, being again available for diffusion and binding to the plasma membrane. Perhaps repeated cycles of this dynamic behaviour would help to the continue formation of active EGFR-containing macropinosomes, which would participate in the spatial propagation of EGF signals in the cell. The precise role of PTPD1 at EGF stimulation sites deserves future research.

## Materials and Methods

### Cell culture and reagents

MCF-7, MDA-MB-231, MDA-MB-468 cells (American Type Culture Collection) and the stable transfectant MCF7-EG [18] were grown at 37°C and 5% (MCF7) or 0% (MDA-MB-231 and MDA-MB-468) CO_2_ in Dulbecco's modified Eagle's medium (DMEM) (Sigma Aldrich, USA), supplemented with 10% inactivated fetal calf serum (FCS) (PAN Biotech GmbH, Germany), 10 mM glutamine (PAN Biotech GmbH, Germany), 100 U/mL penicillin and 100 µg/ml streptomycin (Gibco). Epidermal growth factor (EGF) and EGF-Alexa647 were obtained from Cell signalling and Molecular Probes, respectively. Effectene and Fugene transfection reagents were obtained from Invitrogen and Roche Diagnostics (GmbH), respectively. Paraformaldehyde (PFA) and Triton X-100 were purchased from Sigma-Aldrich and the Fluorolink Cy3 reactive dye from GE Healthcare. Generic anti-phosphotyrosine (PY-72) and anti-tubulin were obtained from In vivo Biotech Services and Sigma-Aldrich, respectively. Anti-E-cadherin and anti-EGFR antibodies were obtained from R & D systems. Rabbit anti-PTPD1 was kindly provided by Dr. Axel Ulrich (Department of Molecular Biology, Max Planck Institute of Biochemistry, Martinsried, Germany). Polyclonal goat-anti-mouse-Ig Alexa-488 was obtained from molecular probes (Eugene, OR). siRNA pools were purchased from Thermo Scientific (Denver, USA).

### Generation of plasmids

A KpnI-PTPD1-AgeI fragment was amplified by PCR with the primers: Forward GGT ACC AAG ATG CCA CTG CCA TTT GGG TTG and Reverse AC CGG TCC GAT GAG CCT GGA GCT TTT CAG G. As template for the PCR reaction we used a pcDNA3.1 vector containing the complete cDNA encoding PTPD1 (kindly provided by Dr Axel Ullrich, Department of Molecular Biology, Max Planck Institute of Biochemistry, Martinsried, Germany). PCR product was cloned in phase with the DNA encoding the mCitrine, GFP or mCherry chromophores (plasmid backbone from Clontech). Mutant lacking FERM domain was generated by amplifying a KpnI-PTPD1DFERM-AgeI fragment by PCR with the primers: Forward CGCGGTACCACCATGTCTCTGCCTAAACCCCAG and Reverse GCGACCGGTGAGATGAGCCTGGAGCTTTTCAG. The PTPD1-mCherry plasmid previously generated was used as template in this reaction. The EGFR-CFP plasmid was previously developed in our laboratory [34], and the Actin-GFP plasmid was previously described [35].

### siRNA and cDNA transfection

PTPD1 down-modulation was obtained by transfection of ON-TARGETplus smart siRNA pools (Thermo Scientific) that contain 4 different siRNA molecules specific for the *PTPN21* gene (we called N21). As a negative control we used non-targeting siRNA pools (Thermo Scientific) (we called NT). 100 nM of N21 specific or NT pools were transfected by using Hiperfect reagent following the manufacturers instructions. Cells were incubated during 48 hours to allow protein down-modulation. Transient transfection of plasmids was performed with the Fugene transfection reagent under manufacturers instructions. Cells were incubated for up to 24 hours to allow the expression of the protein before experiments

### Quantitative western blot

Cells were starved and stimulated or not with 100 ng/ml EGF during the indicated times. In experiments done to check the down-modulation of PTPD1, cells were kept 48 h after siRNA transfection in growing conditions. After incubation times, cells were lysed by 30 min incubation in ice cold RIPA-buffer (50 mM Tris.HCl, pH 7.5, 150 mM NaCl, 5 mM EDTA, 1% Nonidet P-40, 0.5% Na deoxicolate, 0.1% SDS, 1 mg/ml leupeptin, 1 mg/ml pepstatin, 1 mM NaVO_3_, and 1 mM NaF). Cell lysates were further homogenized by using an ultrasonic homogenizer Sonopuls (Bandelin electronic, GmbH, Germany) with 3 cycles of 12 seconds at 40% of maximal power. After centrifugation at 20000×g and 4°C for 30 min to remove the non-soluble material, samples were run in 8% SDS-PAGE and transferred to an Immobilon-FL transfer membrane (Millipore Corporation, USA). Membranes were probed with primary antibodies specific for EGFR, phospho-tyrosine, PTPD1, and tubulin, as indicated. IRDye 800 conjugated donkey anti-mouse and donkey anti-goat, and IRDye 680 conjugated donkey anti-rabbit antibodies (LICOR Biosciences, USA) were used as secondary antibodies. Blots were scanned and fluorescence quantified with an Odyssey Infrared Imager (LICOR Biosciences). Fluorescent signal of PTPD1 was normalised by the tubulin signal. The ratio to the normalised value of NT sample is shown.

### Confocal microscopy

Cells were seeded in LabTek chambered cover glasses (Nunc) coated with 20 µg/ml fibronectin. Live cells were imaged in imaging medium (DMEM without phenol red, PAN Biotech GmbH). Medium was supplemented or not with 10% FCS for growing or starving conditions, respectively. In time lapse confocal microscopy one confocal section was acquired each 30 seconds. 500 ng/ml of EGF-A647 were added to the chambers. Optical sections were acquired with a Leica SP5 confocal scanning laser microscope by exciting specimens at 488, 561, and 633 nm with an Argon, Diode-pumped solid-state (DPSS) and HeNe-633 lasers, respectively. Emission wavelengths accessible to photomultipliers were selected with the Leica software. FRAP experiments were implemented with the Leica SP5 FRAP wizard. In the post bleaching step time-lapse frames were acquired every 10 seconds. Analysis of FRAP data and protein-protein co-localisation was performed with the ImageJ software (NIH, USA). FRAP data are obtained by:
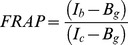



Where *I_b_* is the fluorescence intensity in the bleached area, *I_c_* the fluorescence intensity of the cell and *B_g_* the background in each frame. This calculation corrects for the bleaching of the sample during the experiment. FRAP curves were then obtained by normalising FRAP data by its highest value after bleaching. For analysing the distribution of EGFR, PTPD1 and EGF in endocytic structures, intensity of the background was subtracted, regions of interest in endocytic structures were selected and the intensity in the green (EGFR-GFP), red (PTPD1-mCherry) and far-red (EGF-A647) channel quantified. The ratio between the red and the green fluorescence was calculated for each time point and normalised to the value at time point zero. EGF-A647 signal was normalised to its maximum.

### Confocal FRET-FLIM

Phosphorylation of EGFR was tracked by the FRET-FLIM based method previously developed in our laboratory [36]. Briefly, FRET signal is detected by a decrease in the fluorescence lifetime of the GFP (FRET donor) coupled to EGFR when it is phosphorylated and, consequently, binds the PY72 anti-phosphotyrosine antibody conjugated with the FRET acceptor Cy3. Time correlated single photon counting (TCSPC) images were collected using a LSM Upgrade Kit (PicoQuant, Berlin, Germany) attached to a FV-1000 microscope (Olympus Deutschland GmbH, Hamburg, Germany). A 470 nm pulsed diode laser (LDH 470, PicoQuant, Berlin, Germany) was used as an excitation source. A UMGFPHQ dichroic (Olympus Deutschland GmbH, Hamburg, Germany) and a 500/25 HQ filter were used to detect the emitted photons using a Single Photon Avalanche Photodiode (SPAD). Global tail fit of pixel data was done with the SymPhoTime software (PicoQuant, Berlin, Germany) to generate the lifetime maps. The molar fraction of phosphorylated EGFR-GFP (*α*) was calculated by global analysis of FLIM data as previously developed in our laboratory [19]. Intensity and alpha maps were segmented in single cells by using the CellProfiler freeware. Average values of the intensity (as an estimate of the total amount of receptor) and the <*α*> per cell were then imported in MatLab (Mathworks, UK) for analysing the distribution and the correlation between both parameters. The cumulative distribution of *α* values obtained for the different samples was compared with the Kolmogorov-Smirnov statistical test implemented in Matlab.

### FCS and FCCS

FCS and FCCS were performed with a ConfoCor 3 coupled to a Zeiss LSM 510 laser scanner microscopy with a water immersion Apochromat 40× objective lens, N.A. 1.2 (Carl Zeiss, Germany). PTPD1-GFP or PTPD1-mCherry were transiently transfected in MDA-MB-231, or the MCF7-EG stable transfectants. The low spectral overlapping between GFP and mCherry makes these fluorophores suitable for this approach. GFP and mCherry were excited with the 488 nm line of an argon/2 laser with the powerful set at 50%, and with the 561 nm line of a DPSS-561-10 laser, respectively. The fluorescence emission was filtered through a dichroic beam splitter HFT 405/488/561, and then separated with a secondary beam splitter NFT565 into detection channel 1 (BP615-680) and 2 (BP505-540). The fluorescence was detected by avalanche photodiodes (APDs). 10 experimental autocorrelation functions were acquired for all the measurements done in all the cells analysed, taking the average curve for representation and further analysis. Analysis of FCS and FCCS data is explained in *Methods S1*.

## Supporting Information

Figure S1 **Expression and activation of EGFR-GFP in MCF7-EG cells comparing to MDA-MB-468 cells.** (**a**) WB analysis for EGFR expression. Anti-tubulin antibody is used as a protein load control. (**b**) Time course EGF stimulation in MCF7-EG and MDA-MB-468 cells. WB for anti-phosphotyrosine, EGFR, and tubulin is shown. Numbers indicate time in minutes. (**a** and **b**) Molecular weight markers are used to indicate the correct size of the revealed proteins.
(TIF)

Figure S2 **Distribution of EGFR and PTPD1 in MCF7 cells prior to the stimulation with EGF tracked in movies S1 and S2.** The ROI of interest shown in figure 2 is located by a white square.
(TIF)

Figure S3 **(a) Co-localisation of PTPD1-mCherry and actin-GFP transfected in MDA-MB-231 cells.** Red, green, merged, and transmission channel are shown. Co-localisation histograms of ROIs and the cell are shown (lower panels) (**b**) Distribution of EGF and actin during EGF stimulation of MDA-MB-468. Actin and membrane dynamics precede the formation of EGF containing vesicles where actin is not detected. Numbers indicate the times of shown frames in minutes and seconds.
(TIF)

Figure S4 **FCCS positive control.** Figure shows blue lines of Auto and crosscorrelation functions obtained with the fluorescent fusion protein mCherry-p38-GFP transfected in MCF7 cells. Theoretical fits (red, green and black lines over experimental data) and the corresponding residuals (lower graph) are shown.
(TIF)

Methods S1 (DOC)

Movie S1 **Dynamics of PTPD1 during stimulation of MCF7 cells with EGF.** Live cells transfected with PTPD1-mCherry and EGFR-EG where tracked by time-lapse confocal microscopy before and during stimulation with EGF-A647. Elapsed time 30 seconds.
(MOV)

Movie S2 **Zoom of the ROI in the cell represented in figure S2 and tracked in movie S1.**
(MOV)

Movie S3 **Dynamics of acting during stimulation of MDA-MB-468 cells with EGF-A647.** Time-lapse confocal microscopy with an elapsed time of 30 seconds is shown.
(MOV)
