# Supplementary figures and images for: Dynamic Recruitment of Protein Tyrosine Phosphatase PTPD1 to EGF Stimulation Sites Potentiates EGFR Activation
Source: PLoS One. 2014 Jul 25;9(7):e103203. doi: 10.1371/journal.pone.0103203 (PMC4111557; doi:10.1371/journal.pone.0103203)

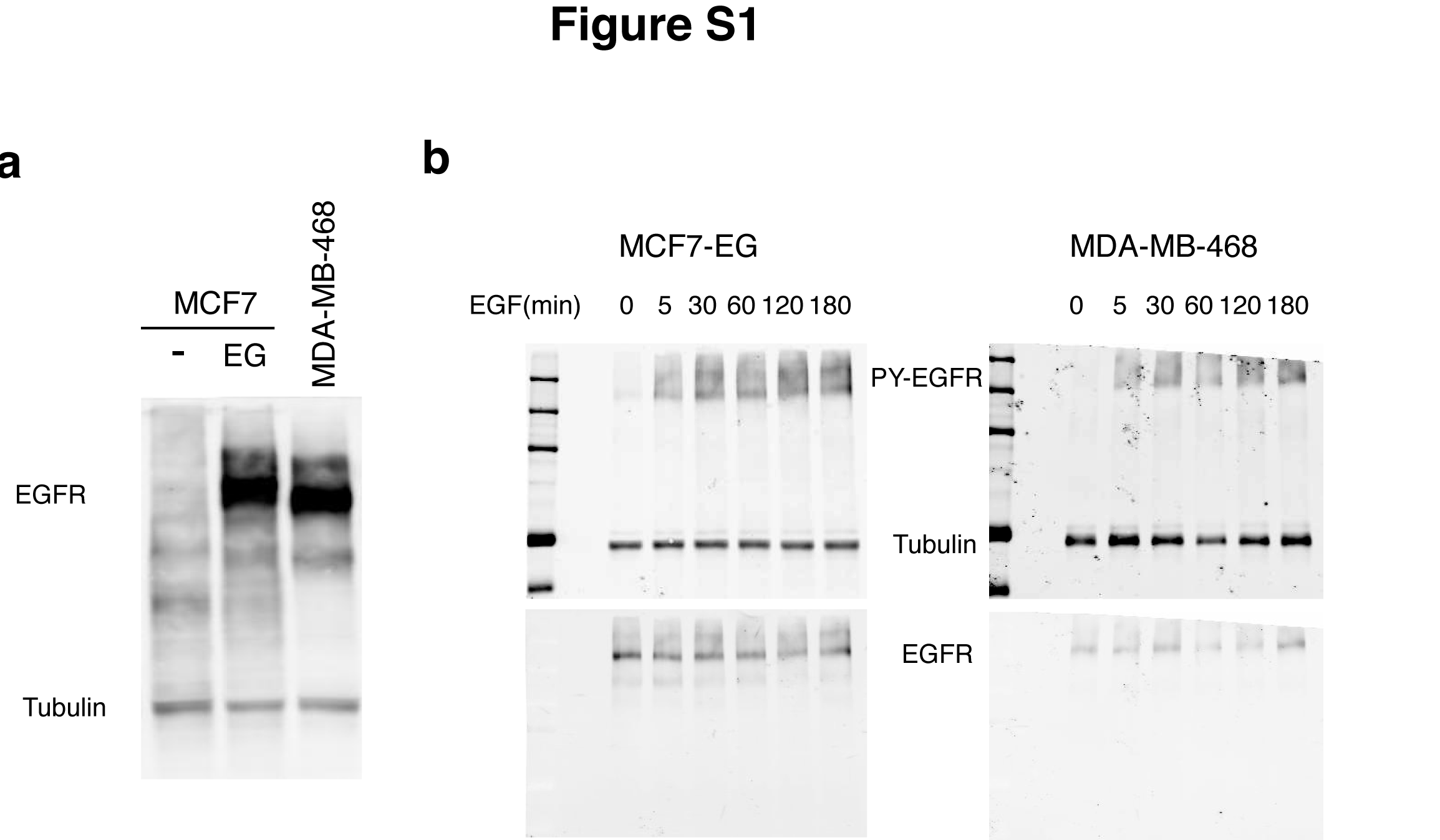

Supplement: Figure S1 — Expression and activation of EGFR-GFP in MCF7-EG cells comparing to MDA-MB-468 cells. (a) WB analysis for EGFR expression. Anti-tubulin antibody is used as a protein load control. (b) Time course EGF stimulation in MCF7-EG and MDA-MB-468 cells. WB for anti-phosphotyrosine, EGFR, and tubulin is shown. Numbers indicate time in minutes. (a and b) Molecular weight markers are used to indicate the correct size of the revealed proteins. (TIF) [file pone.0103203.s001.tif]

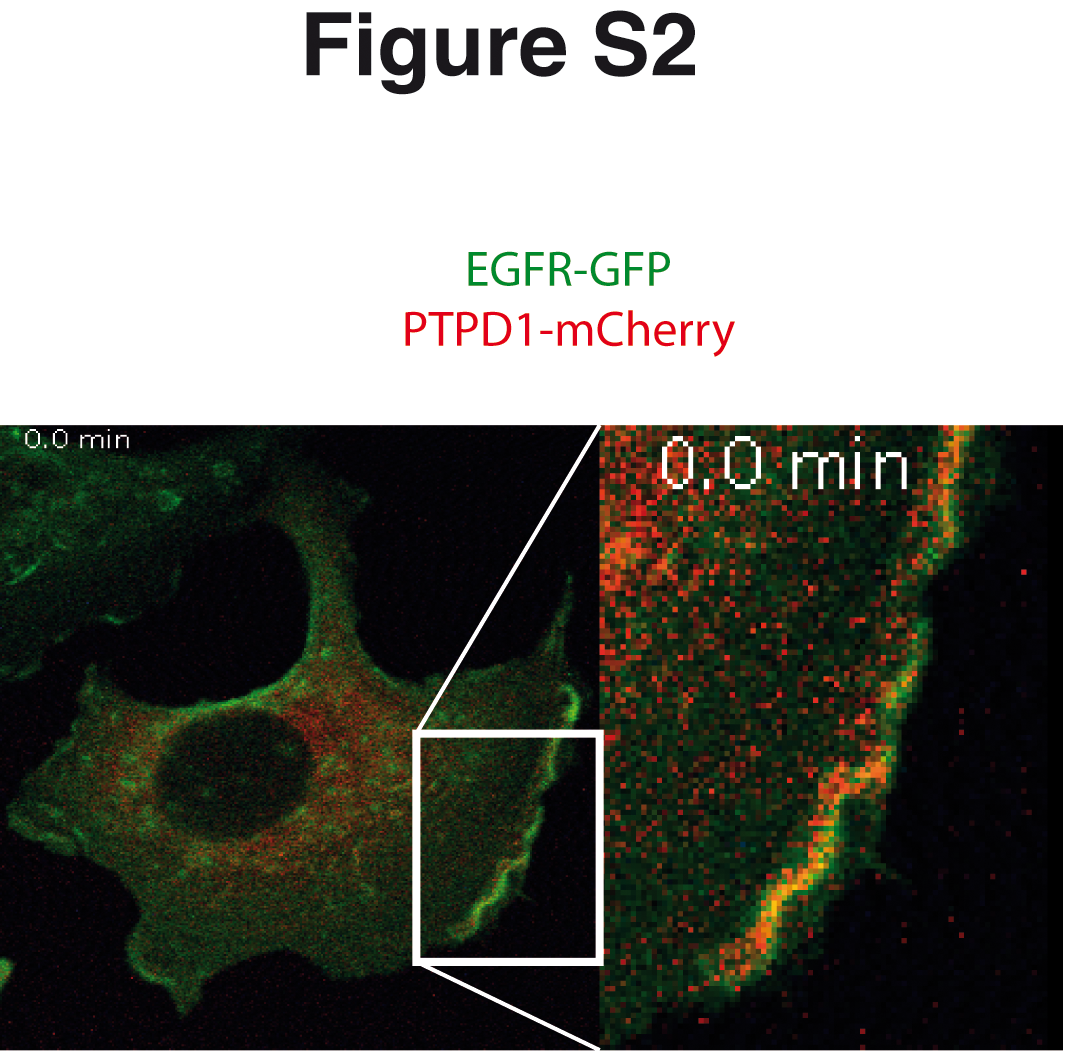

Supplement: Figure S2 — Distribution of EGFR and PTPD1 in MCF7 cells prior to the stimulation with EGF tracked in movies S1 and S2. The ROI of interest shown in figure 2 is located by a white square. (TIF) [file pone.0103203.s002.tif]

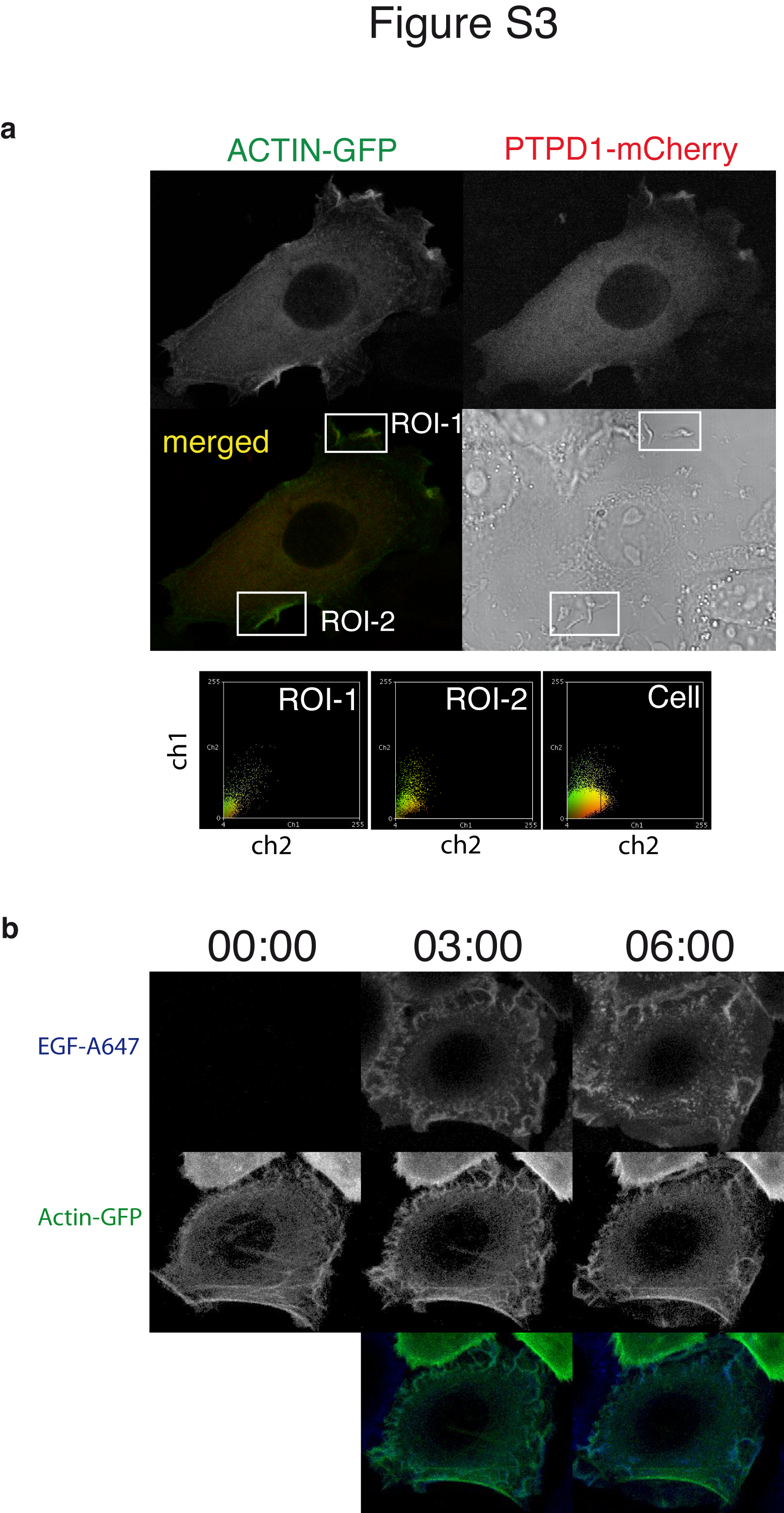

Supplement: Figure S3 — (a) Co-localisation of PTPD1-mCherry and actin-GFP transfected in MDA-MB-231 cells. Red, green, merged, and transmission channel are shown. Co-localisation histograms of ROIs and the cell are shown (lower panels) (b) Distribution of EGF and actin during EGF stimulation of MDA-MB-468. Actin and membrane dynamics precede the formation of EGF containing vesicles where actin is not detected. Numbers indicate the times of shown frames in minutes and seconds. (TIF) [file pone.0103203.s003.tif]

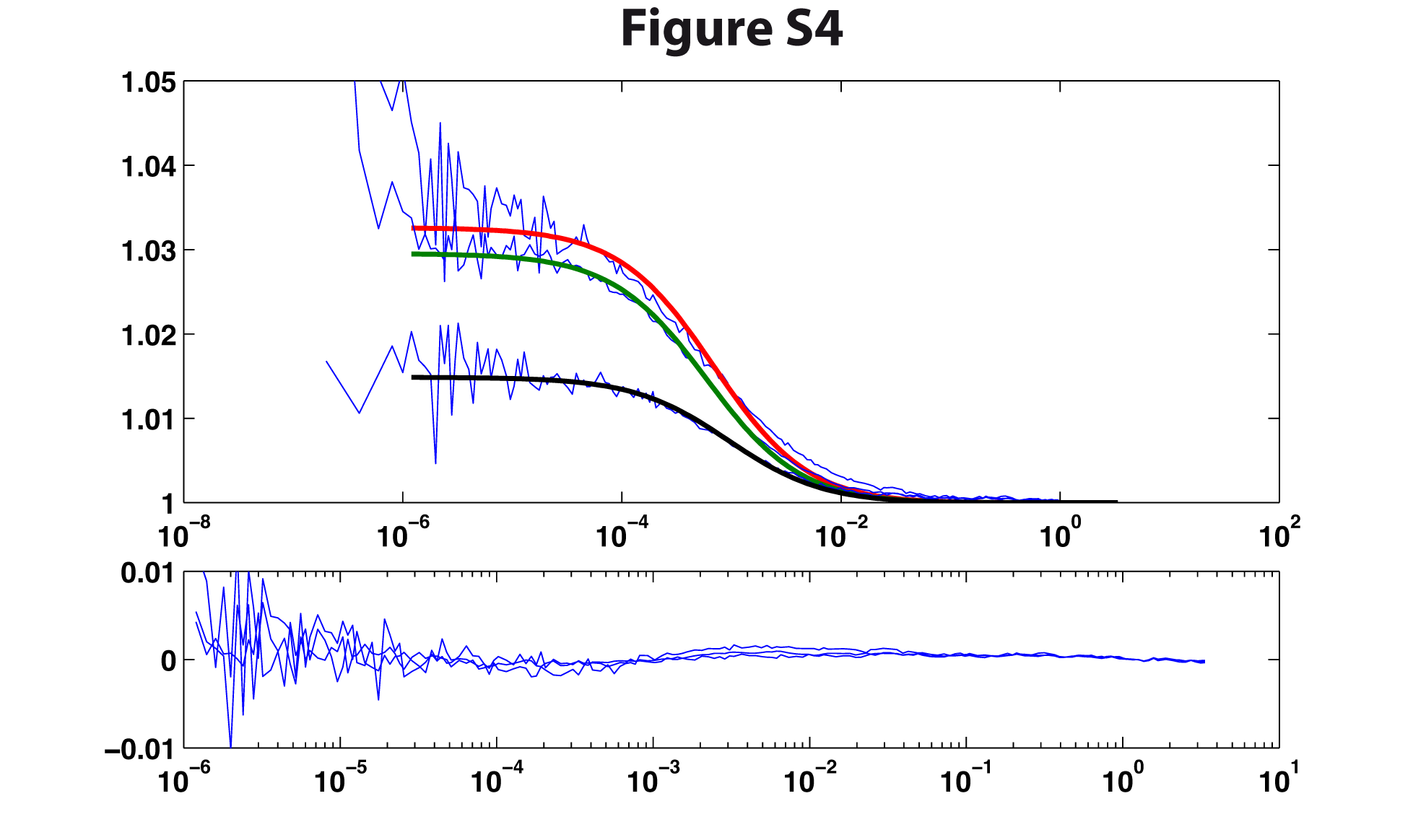

Supplement: Figure S4 — FCCS positive control. Figure shows blue lines of Auto and crosscorrelation functions obtained with the fluorescent fusion protein mCherry-p38-GFP transfected in MCF7 cells. Theoretical fits (red, green and black lines over experimental data) and the corresponding residuals (lower graph) are shown. (TIF) [file pone.0103203.s004.tif]
