# Supplementary material for: Dynamic Recruitment of Protein Tyrosine Phosphatase PTPD1 to EGF Stimulation Sites Potentiates EGFR Activation
Source: PLoS One. 2014 Jul 25;9(7):e103203. doi: 10.1371/journal.pone.0103203 (PMC4111557; doi:10.1371/journal.pone.0103203)
Supplement: Methods S1 — (DOC) [file pone.0103203.s005.doc]

**Methods S1**

FCS and FCCS analysis

Experimental autocorrelation (*G_ij_*) was generated from sections of the intensity traces that do not show any intensity fluctuation due to photobleaching or cellular movement:

$G_{ij}(\tau)=1+\frac{\langle\delta I(t)\delta I(t+\tau)\rangle}{\langle I^{2}\rangle}$ (1)

Where *I*(*t*) is the fluorescence intensity measured at time *t*, and *I*(*t*+*τ*) the fluorescence intensity measured after the delay time *τ*. We then fitted the autocorrelation functions (ACFs) between 10 μs and 1s according to different diffusion models. ACFs of PTPD1-mCherry and PTPD1-GFP in figure 3a were fitted to a free 3D diffusion, where the translational diffusion (*τ_d_*) depends on the shape of the confocal volume (*s*), which is defined as the ratio of the axial *ω_z_* and radial *ω_xy_* axes (*s*=*ω_z_*/*ω_xy_*). ACFs were then fitted to:

$G_{ij}(\tau)=\frac{1}{N}\cdot\frac{1}{\left(1+\frac{\tau}{\tau_{d}}\right)\cdot\sqrt{1+\frac{\tau}{s^{2}\cdot\tau_{d}}}}+1$ (2)

Also ACFs of PTPD1-mCherry and PTPD1-GFP were fitted to an anomalous 2D diffusion model :

$Gij(\tau)=\frac{1}{N}\cdot\frac{1}{\left(1+\left(\frac{\tau}{\tau_{d}}\right)^{a}\right)}+1$ (3)

Where *a*, ranging between 0 and 1, represents the anomalous exponent that indicates the sub-diffusive state of the protein. In this model, the diffusion coefficient can be calculated as

$D=\frac{\omega_{xy}^{2}}{4\tau_{d}^{2}}$ (4)

The same model was used for fitting cross-correlation curves.

In both models *N* indicates the number of particles in the confocal volume calculated from *G_ij_*(0)=1/*N*+1.

EGFR-GFP autocorrelation curves were fitted with a function with a free 3D diffusion model for the fast component and an anomalous 2D diffusion for the slow component:

$Gij(\tau)=\frac{1}{N}\cdot\left(\frac{1}{\left(1+\frac{\tau}{\tau_{d_{1}}}\right)\cdot\sqrt{1+\frac{\tau}{s^{2}\cdot\tau_{d_{1}}}}}+\frac{1}{\left(1+\frac{\tau}{\tau_{d_{2}}}\right)^{a}}\right)+1$ (5)

In order to check the overlap between the detection volumes, auto and cross-correlation curves of GFP-p38-mCherry were fitted to a 3D free diffusion model as in equation 2. The fraction of PTPD1 in complex with EGFR cannot be accurately calculated due to the presence of an unknown amount of endogenous PTPD1 in the cell.

**References**

1. Banks DS, Fradin C (2005) Anomalous diffusion of proteins due to molecular crowding. Biophys J 89: 2960-2971.
